# Effect of Copper Segregation at Low-Angle Grain Boundaries on the Mechanisms of Plastic Relaxation in Nanocrystalline Aluminum: An Atomistic Study

**DOI:** 10.3390/ma16083091

**Published:** 2023-04-13

**Authors:** Vasiliy Krasnikov, Alexander Mayer, Polina Bezborodova, Marat Gazizov

**Affiliations:** 1 Physical Department, Chelyabinsk State University, Chelyabinsk 454001, Russia; 2 Laboratory of Mechanical Properties of Nanoscale Materials and Superalloys, Belgorod State University, Belgorod 308015, Russia

**Keywords:** symmetrical tilt low-angle grain boundary, grain boundary slip, dislocation, grain rotation, Al-Cu alloy, molecular dynamics, shear strength

## Abstract

The paper studies the mechanisms of plastic relaxation and mechanical response depending on the concentration of Cu atoms at grain boundaries (GBs) in nanocrystalline aluminum with molecular dynamics simulations. A nonmonotonic dependence of the critical resolved shear stress on the Cu content at GBs is shown. This nonmonotonic dependence is related to the change in plastic relaxation mechanisms at GBs. At a low Cu content, GBs slip as dislocation walls, whereas an increase in Cu content involves a dislocation emission from GBs and grain rotation with GB sliding.

## 1. Introduction

Grain boundary engineering controlled by chemistry and thermomechanical processing (TMP) routes has been considered a key approach to improve the macroscopic mechanical properties of nanostructured metallic materials [1,2,3,4]. Low-angle grain boundaries (LAGBs) with misorientation angles *θ* of less than 15° are known to form in various polycrystalline metallic materials fabricated by TMP, including severe plastic deformation at intermediate temperatures [3,4]. Note that a large density of these LAGBs may hinder plastic deformation and improve the strength of these materials [2,3,4]. Thus, knowledge of unambiguous relationships between the microstructure and macroscopic mechanical behavior, as well as their features related with alloying, is important to design new approaches for strengthening [1,2,3,4,5,6,7,8]. However, the microstructural analysis of empirical results related with a phenomenon does not provide a deep insight into these fundamental processes to adjust mechanical properties more accurately. In this case, the molecular dynamics (MD) method can be utilized, providing a true picture of the dynamic peculiarities in the mechanical behavior of the system [9], especially in the context of the features of load- and time-dependent relaxation processes [10]. The processes associated with LAGB mobility are studied using molecular dynamics [11,12,13]. It has been shown in the works [11,12] that the LAGB slip is accompanied by the annihilation of dislocation walls, which leads to the coarsening of nanocrystalline (NC) grains. The paper [13] demonstrates that, depending on the misorientation angle *θ*, the mechanism of the grain boundaries (GB) motion changes from sliding to slip.

In aluminum alloys, Cu is known to be an important alloying element demonstrating a prominent strengthening effect by the forming of intermetallic particles [3,4,5,6,7,8]. However, this element also tends to segregate at lattice dislocations and grain boundaries [3,4,5,7]. The effect of the alloying atom segregation on the properties of GBs and their contribution to the mechanical properties of aluminum alloys have been intensely studied in [4,7,14,15,16,17]. This effect is studied with MD in [14,15] considering the effect of Mg segregation. The Ab initio works of the segregated atom’s role on the behavior of GBs are also actively developed [16,17]; however, such an approach is strictly limited in space and time scales.

In the present work, the plastic relaxation mechanisms and mechanical response depending on the Cu concentration at the GB in NC aluminum has been investigated by MD simulations. The LAGB with an *θ* of ~12° around the tilt axis of <110>_Al_ and entirely comprising of periodically, planarly arranged lattice defects—edge dislocations—was used as a research object. Note that this LAGB can be classified as a symmetric tilt grain boundary of type Σ99a <110>_Al_ in the coincidence site lattice nomenclature [18]. An extensive MD-based analysis has been performed to characterize the behavior of dislocation boundaries with Cu segregation in dilute Al-Cu alloys.

## 2. MD Problem Statement

### 2.1. Creation of MD System with GBs

The system containing symmetric tilt LAGBs was created using the Atomsk software package [19]. The dimensions of the system along the *x*-, *y*-, and *z*-axis are 100 nm, 23 nm, and 33 nm, correspondingly. The system contains 4,632,320 Al atoms. Periodical boundary conditions are applied along all axes of the system. 

**Figure 1:**
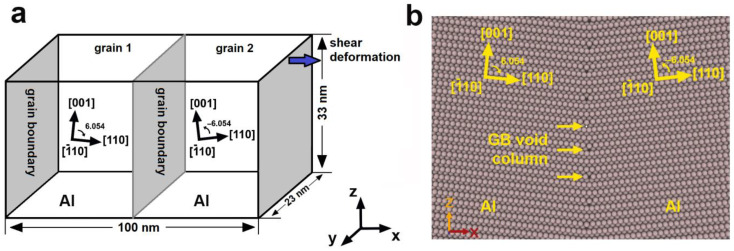
(**a**) Schematic representation of the system containing symmetrical tilt LAGB; (**b**) an atomic structure formed at symmetrical tilt LAGB with a misorientation angle of 12.108°. Periodically located columns of voids at the GB are visible.

The system with two GBs was created using the Atomsk software in accordance with the scheme demonstrated in Figure 1a. The following procedure was applied. First, two nodes are specified at the reduced coordinates (0.25; 0.5; 0.5) and (0.75; 0.5; 0.5) of the corresponding system’s dimensions. Second, the seeds of aluminum lattice are inserted at the node positions so that the crystallographic directions of the lattice form the following configuration: $\left[\bar{1}10\right]$ and [001] directions coincide with the *y-* and *z*-axes of the system and the [110] direction is rotated by an angle of 6.054° clockwise in the left part, and by −6.054° in the right part of the system. Third, the lattice seeds are duplicated in all directions and cut at the GBs. This procedure leads to the formation of two identical LAGBs. The LAGB formed in this case refers to a symmetric tilt low-angle grain boundary with a misorientation angle of 12.108° close to a special type Σ99a (110)_Al_, taking into account the Brandon criterion $\Delta \theta =\theta \cdot \Sigma^{-1/2}\approx \pm 1.2{}^{\circ}$ [18]. The resulting arrangement of atoms at the GB is shown in Figure 1b. The distribution of atoms shows columns of voids belonging to GBs surrounded by five Al atoms.

### 2.2. Energetically Preferable Positions of Cu Atoms at GBs

Energetically preferable positions of Cu atoms at the GB are determined with the procedure of energy minimization performed with molecular dynamics simulations. Classical molecular dynamics is performed with the LAMMPS package [20]. Interactions of atoms are described with an angle-dependent potential (ADP) by Apostol and Mishin [21], which is widely used in the description of the Al-Cu alloy’s properties [22,23,24,25,26], since it reproduces with a sufficient accuracy the elastic constants and energies of various defects in both these metals and their intermetallic phases. In addition, this potential is rather resource-efficient, allowing one to use it in MD simulations of systems containing millions of atoms. 

The system described in Section 2.1 is used as the initial distribution of Al atoms, in which one Cu atom is added. A pentagonal void is used as a central point, around which the Cu atom is placed randomly in a parallelepiped with dimensions of 1.2–2.4 nm along the *x*-axis, 0.286 nm along the *y*-axis, and 1.5 nm along the *z*-axis. The choice of size along the *x*-axis is associated with the Cu position search in Al lattice, where the influence of the GB ceases to be felt; along the *z*-axis with the periodicity of the GB voids arrangement; and along the *y*-axis with the Al lattice periodicity in the $\left[\bar{1}10\right]$ direction. After placing the Cu atom, the system energy is minimized, and the output energy value is further used to analyze the most preferable location of Cu atoms in the system. 

**Figure 2:**
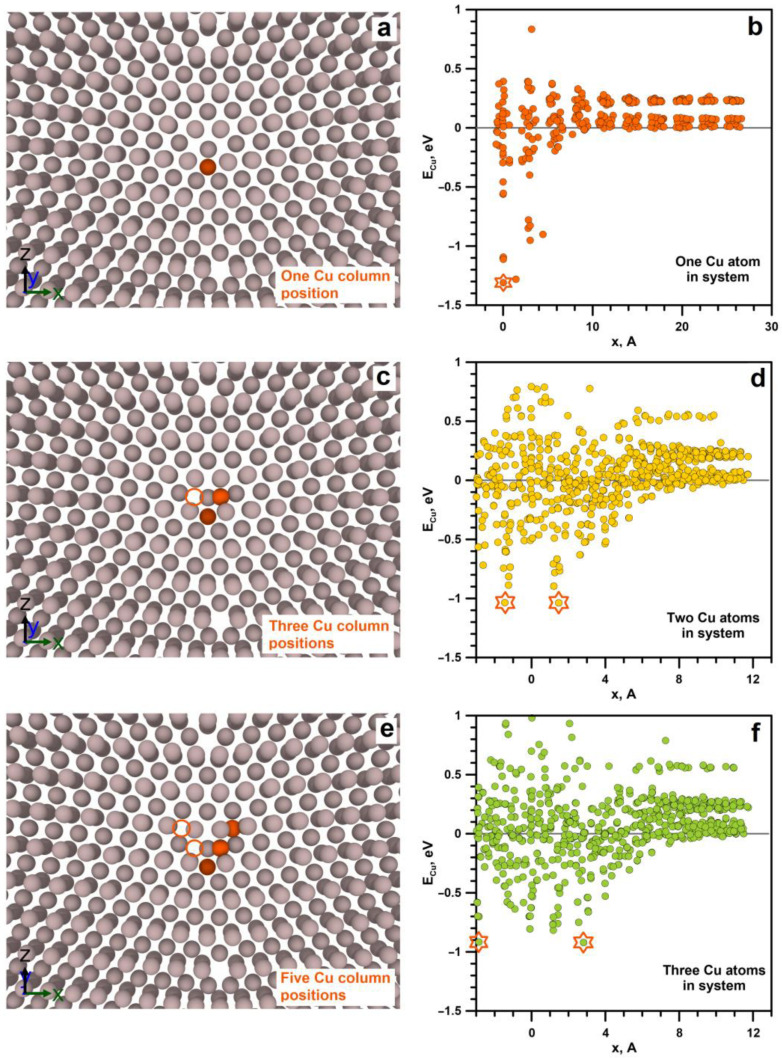
(**a**,**c**,**e**) Positions of Cu atoms at GB. The filled orange circles correspond to the Cu atom with the calculated minimal energy; the contour orange circles are symmetrical with respect to GB reflections of the calculated positions of the Cu atoms. Grey circles are Al atoms. (**b**,**d**,**f**) Dependencies of energy on the Cu atom position in the system near GB after energy minimization. The asterisks indicate the minimum energy of Cu atoms in the system by which the Cu atom positions at GB are determined. All positions of Cu atom are plotted in the *xz*-plane. *x* = 0 corresponds to Cu atom position at GB void center.

The energy of the system with the Cu atom placed at a distance of more than 1 nm reaches several stationary levels (Figure 2b); the energy with the minimal value is taken as a base level. All energy values, which correspond to each Cu atom’s position in the system, are plotted.

The position of the first Cu atom in the system with minimal energy is in the pentagonal GB void center (Figure 2a). The value of the Cu atom’s energy is found in this position to be −1.31 eV (Figure 2b) compared to the minimal energy of the Cu atom at the distance of 1.2–2.4 nm along the *x*-axis from GB, where no GB influence is observed.

The procedure for finding the first Cu atom position is further repeated for the second and third Cu atoms in the system. The second Cu atom is added to the system where the first Cu atom is already added in a pentagonal void; after that, the energy of the system is minimized. In this case, the length of the parallelepiped along the *x*-axis is shortened to 1.2 nm because, in the case of the first Cu atom, it is seen that the influence of the boundary on the energy levels in aluminum disappears at the distance of about 1 nm (Figure 2b). The energy distribution along the *x*-axis in this case has two minimums on opposite sides of the pentagonal void with the value of −1.03 eV (Figure 2e). The position of the second atom relative to the pentagonal pore is shown in Figure 2c.

The search for the position of the third Cu atom is carried out according to a similar procedure with the first and second Cu atoms added to the system. The minimum energy value for the third Cu atom in the system is found to be −0.92 eV (Figure 2f); the corresponding position of the Cu atom is shown in Figure 2e.

By a direct calculation of the minimal Cu atom’s energy after the addition of the next atom, the positions of copper atoms are determined on the right of the pentagonal GB void. Positions symmetric to them on the left from the GB are not directly calculated by adding fourth and fifth atoms to the system, although they are present in the dependence of the Cu atom’s energy on the *x*-coordinate, obtained during calculations for the second and third Cu atoms.

The results of MD calculations show that Cu atoms tend to segregate on the LAGBs in aluminum due to their significantly lower energy compared to pure Al remote from GB. The tendency for Cu to accumulate at the GBs of NC aluminum has been observed in a number of experimental works [27,28,29,30,31,32,33]. Previously, the Cu atom’s energy at the GB was calculated by the authors [17] using ab initio calculations. The calculated energy of the Cu atom at the GB turned out to lie in the range from −0.494 to −0.587 eV. The energies of the Cu atoms at the GB determined in our work are of the order of −1 eV. The difference in the values can be explained by the fact that systems in ab initio calculations contain a small atom number of about several tens and are periodic in all directions, which ensures Cu atoms’ mutual influence. In our calculations, the first Cu atom is placed in the system containing only Al atoms, the number of which is 4,632,320, that exclude Cu atoms mutual influence through periodic conditions. At the same time, the addition of the second and third Cu atoms to our system leads to some increase in their energy. Therefore, the results of ab initio calculations serve as an upper estimate of the energy for Cu atoms periodically located along the boundary.

### 2.3. MD Simulation of Shear Deformation System with Cu Segregation at GBs

To investigate the effect of various Cu content at GBs, the successive filling of columns along the $\left[\bar{1}10\right]$ direction with positions of Cu atoms equivalent to positions with three minimum energies determined in Section 2.2 is realized. First, we fill each pentagonal void column at both GBs by the Cu atoms in the position supposed to be located periodically along the $\left[\bar{1}10\right]$ direction with the minimum energy of −1.31 eV identified in Section 2.2. The placement of the Cu atom in each position along the column is carried out in order to satisfy the given fraction of atoms from the possible complete filling of the column. The filling fractions of all columns per each GB are chosen to be the same. The correspondence of the filling fractions with the surface density of Cu atoms segregated at the GB is given in Table 1.

Simultaneous filling in the second and third columns, positions for which correspond to the second energy minimum of −1.03 eV and are shown in Figure 2c, is carried out after the complete filling of the columns in the first energy minimum Cu atom positions. In this case, the placement of the Cu atom in each position of both columns is performed independently in order to satisfy a given fraction of the complete filling of the column. 

**Figure 3:**
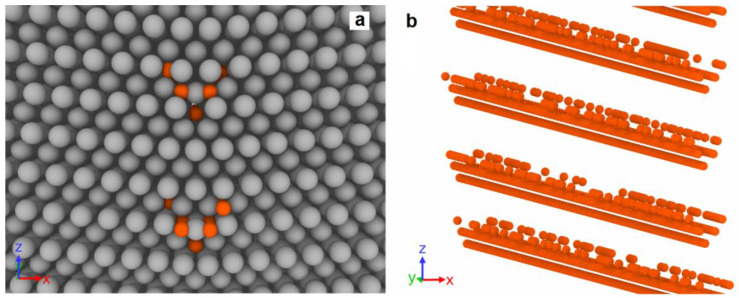
Initial view of the system with five columns of Cu atoms located near each pentagonal void at GB. The fourth and fifth columns of Cu atoms are 15% filled. (**a**) *xz*-plane of system; (**b**) perspective view of Cu columns. Grey circles are Al atoms, orange—Cu atoms.

Similarly, the filling of the fourth and fifth columns with the energy value of −0.95 eV occurs after the complete filling of the second and third columns. An example of the system with completely filled first, second, and third columns and partially filled fourth and fifth columns is shown in Figure 3. The degree of filling of the fourth and fifth columns corresponds to 15%.

The upper limit of the range of copper content per GB considered by us (corresponds to 100% filling of 5 columns), recalculated for the Cu concentration in a parallelepiped with the height of 1 nm and unit surface, can be estimated as 13 at.%. This estimation falls within the experimentally measured Cu concentration at GB in Al–Cu–Mg alloy (A2024) processed by high-pressure torsion [32].

After the procedure of Cu atom placement at GBs, we obtained a set of systems with a different Cu content. The full number of systems used for MD simulations can be seen in Table 1. Additionally, the system without the Cu content is simulated.

Before the MD simulation of the system’s shear deformation, the systems are heated up to 300 K for 10 ps and then kept in a thermostat at the constant temperature of 300 K and a barostat with all zero components of the stress tensor for 100 ps. Further, a uniform shear deformation $\varepsilon_{xz}$ is applied to the systems using the standard LAMMPS command “fix deform”. This command rescales atom coordinates and MD box inclination, which is physically equivalent to a simple shear of the whole MD system by inertia and eliminates boundary effects. The strain rate is set equal to ${\dot{\varepsilon }}_{xz}=\left(1/2\right)\left(\partial u_{x}/\partial z\right)=1.635\cdot 10^{8}$ s^−1^, where $u_{x}$ is the displacement in the x direction. The temperature is kept constant and equal to 300 K during the deformation.

The analysis and visualization of the resulting atomic distributions are performed using the Ovito software package [34]. The defect structure is analyzed by calculating the centrally symmetric parameter [35]. For analysis, the time dependence of an atom number with centrally symmetric parameters exceeding five is obtained. The dislocation structure is identified using the dislocation extraction algorithm (DXA) [36]. For all the atoms of the system, the displacement vectors are calculated, and the displacements associated with the overall deformation of the system are subtracted. Further, 100 atoms with the maximum vertical displacement are selected, and the value of the maximum vertical displacement is calculated by averaging their displacements. The mechanical response of the system to the applied deformation is recorded on the basis of the shear stresses $\sigma_{xz}$ averaged over the system.

## 3. Plastic Relaxation Mechanisms and Stress State of MD Systems

Deformation of the system leads to the occurrence of $\sigma_{xz}$ shear stress. Shear stresses act on the GBs, activating plastic relaxation in the system. Depending on the Cu content, different mechanisms of plastic relaxation have been identified.

### 3.1. GB Slip

**Figure 4:**
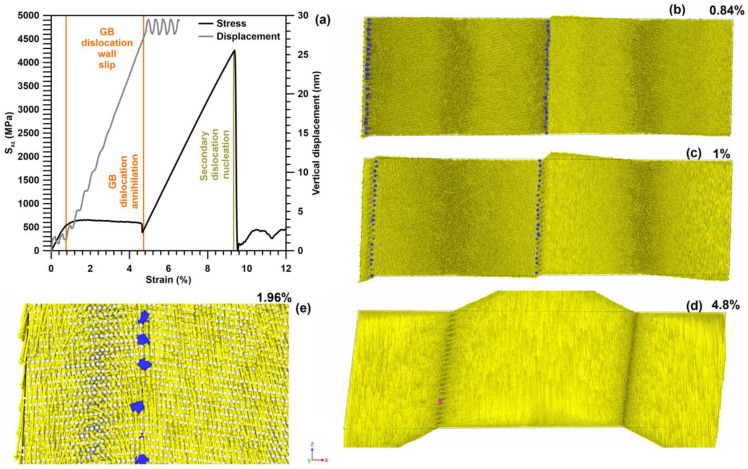
(**a**) Stress–strain dependence and dependence of vertical displacement in the system on strain. (**b**,**c**) Displacement vectors of atoms (yellow) and dislocation walls (blue dots). Dislocation walls slip to each other. The movement of dislocation walls leads to rearrangement of the atomic structure, causing a change in the position of atoms along *z*-axis. (**d**) Annihilation of the dislocation wall. A single stair rod segment (purple dot) is visible at the annihilation place. (**e**) Continuous distribution of displacement vectors forming vortex structures behind the left GB slipping to the right.

In the absence of Cu atoms at GBs, the applied shear stress leads to the slip of GBs as whole dislocation walls towards each other. Dislocations in the wall are predominantly perfect dislocations; the fraction of other segments is of about 2.2%. Previously, such a motion was studied in detail in [11,12]. At the beginning of GB slip, the dependence of shear stress on deformation deviates from the linear function of the strain (Figure 4a). The onset of plastic flow occurs at a shear stress of about 550 MPa. An increase in the dislocation wall velocity leads to the shear stress reaching a plateau with a level close to constant at 600–650 MPa, which is maintained until the moment when the two dislocation walls meet each other. The slip of GBs is accompanied by the rearrangement of the atomic structure, since the atomic planes (001) are tilted in opposite directions on the opposite side of the GBs. This rearrangement of the atomic structure after GB passage provokes atom vertical displacement, which is clearly seen in Figure 4b–d. The vertical displacement of atoms on opposite sides of the slipping GBs happens in different directions. For both boundaries, the atoms located on the right are displaced along the *z*-axis, and on the left, against the *z*-axis. The conjugation of oppositely directed vertical displacements occurs near moving GBs through clearly distinguishable vortex-like structures of displacement vectors (Figure 4e). The maximal atom vertical displacement is observed in the places of the GBs initial location. With increasing shear strain in the system, an increase in the maximum vertical displacement is observed (Figure 4a). 

When the dislocation walls approach each other, their movement is accelerated by the mutual action of elastic fields; the rate of plastic relaxation increases, and a slight drop forms on the stress–strain dependence (Figure 4a before strain 0.12). The meeting of the dislocations of opposite signs leads to their annihilation, after which no mobile dislocations remain in the system, and only individual stair rod dislocations are observed at the site of dislocation wall annihilation (Figure 4d). After GB dislocation wall annihilation, the vertical motion of atoms in the system stops, and the dependence of the vertical displacement on the deformation reaches a near-stationary level.

The absence of mobile dislocations in the system stops plastic relaxation that leads to a new increase in shear stresses in the system according to a linear law. This growth is completed at the shear stress of 4250 MPa only after the nucleation of secondary dislocations occurs in the system. Such a high value is observed because the magnitude of the stress acting on the planes of the $\left[\bar{1}10\right]\;\left(111\right)$ slipsystem typical for FCC metals is significantly lower when projected using the Schmidt factor. The nucleation of secondary dislocations leads to a sharp drop in stresses in the system (Figure 4a). The dislocation sources are activated in those places where the stair rod dislocations were formed. The nucleation of secondary dislocations occurs on several planes of the slip system; therefore, further plastic deformation is accompanied by the intersection of dislocations and their heterogeneous multiplication. Therefore, a significant increase in shear stress in the system is no longer observed.

**Figure 5:**
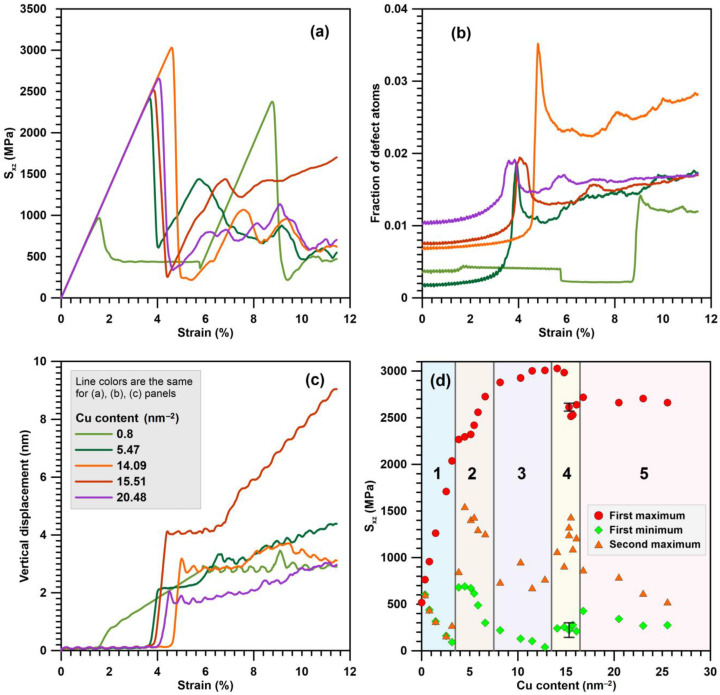
(**a**) Stress–strain dependencies, (**b**) dependencies of the defect atom fraction on the strain and (**c**) dependencies of maximal atom displacement. (**d**) Dependencies of the critical resolved shear stress of the first minimum and the second maximum at stress–strain curves on Cu content at GBs. Area “1” corresponds to plastic relaxation due to GB slip as dislocation walls. In area “2”, plastic relaxation occurs due to grain rotation. In areas “3” and “5”, plastic relaxation occurs due to GB and intragranular dislocation processes. In area “4”, GB dislocation emission stimulates sliding of grains along one GB with rotation of the system as a whole. The error bars mark the standard deviation in the results of 5 calculations for a system with 100% filling of three Cu columns (Cu content is 15.32 nm^−2^).

**Figure 6:**
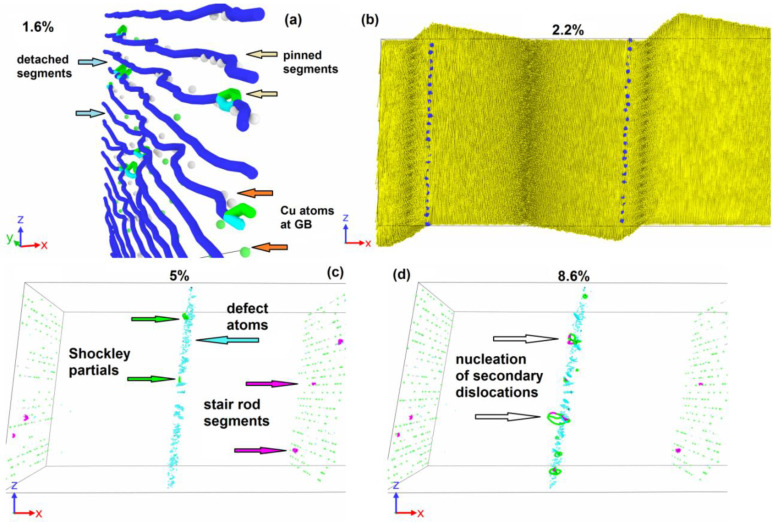
Deformation of system with 15% filling of one column by Cu atoms (0.81 nm^−2^) in each pentagonal void at GBs. (**a**) GB representing dislocation wall pinned at Cu atoms (white and green circles). (**b**) Displacement vectors of atoms (yellow) and dislocation walls. Dislocation walls slip to each other. (**c**) Place of dislocation wall annihilation. Light-blue points are defect atom clusters; green points are Cu atoms in the original places of GBs. (**d**) Nucleation of secondary dislocations from defect atom clusters. Dislocation structure is determined by DXA: blue lines are perfect dislocations, green—Shockley partials, light blue—Frank partials, purple—stair rod segments.

The introduction of Cu atoms to GBs provokes a significant change in the stress behavior with the course of deformation in the systems (Figure 5a). At low Cu concentrations, individual dislocations of the dislocation wall are pinned at the GBs. Figure 6 shows the evolution of a system containing one column of Cu atoms with 15% filling inside each pentagonal void at GBs. The displacement of dislocations is activated in places where Cu atoms are missing in the columns (Figure 6a), while the dislocation segments that touch Cu atoms remain immobilized. The complete detachment of the dislocation wall from Cu atoms occurs later than the beginning of GB motion in pure aluminum. While the dislocation wall remains pinned, the stresses in the system rise with a constant slope that leads to the formation of a sharp peak after the dislocation wall is detached (Figure 5a). For the system with 15% one column filling, the first stress peak is about 950 MPa. After the beginning of the GB slip, the further evolution of stress during the deformation resembles a pure aluminum case. The dislocation walls enter a stationary mode of motion, and the plastic relaxation rate is compared with the external deformation rate; therefore, a close to constant stress level of 450 MPa is established in the system (Figure 5a). Since the movement of the dislocation walls begins later in this case and higher shear stresses are reached, the dislocations are accelerated to higher velocities which ensures a lower stress level on the plateau compared to the pure aluminum case. A constant level of stresses is maintained in the system until the dislocation walls’ meeting and annihilation. After the beginning of GB movement in the system, similarly to pure aluminum case, the atom vertical movement is activated (Figure 6b) that is associated with the atom displacement during GB passage. After GB detachment from Cu atoms, a certain number of defects in the form of stair rod dislocations remain on them (Figure 6c), in contrast to the pure aluminum case. Similar to the pure aluminum case, a certain amount of defect atoms is formed at the site of GB annihilation (Figure 6c); however, their number is higher in the case of a system with Cu atoms. This is due to the fact that when dislocations are separated from Cu atoms, the dislocation lines in the walls are split, and at the same strain of 10.6% close to the moment of GB annihilation, the part of imperfect segments increases to 3.5% for 15% filling of one column system, while in pure aluminum it is 2.2%. 

After the dislocation walls annihilate, further deformation leads to a linear stress increase in the system since there are no mobile defects for plastic deformation. The linear increase in stress in the system ends at the value of about 2350 MPa, which is much lower than the case of pure aluminum. Such a decrease in the nucleation stress is associated with a significantly larger defect atom number in the area of GB annihilation, which is formed during the split dislocation annihilation. Defect atom clusters become places for the secondary dislocation nucleation in the system, essentially lowering the nucleation threshold (Figure 6d).

The dependencies of the defect atom fraction in the system (Figure 5b) and the maximum vertical displacement (Figure 5c) on deformation demonstrate clearly distinguishable areas of initial rest of the GBs until strain reaches 1.8%. After this strain, the dislocation walls are detached from Cu atoms and the number of defect atoms somewhat increases, since, in addition to moving dislocations, defect atoms remain in the system around Cu atoms. The defect atom fraction in the system remains approximately constant as long as the GBs slip, and the vertical displacement increases with a constant rate. After the meeting and annihilation of GBs, the defect atom number in the system decreases, and the vertical displacement stops. Both these dependencies remain at approximately the same level until reaching 8.6% strain. The next sharp increase in the defect atom number occurs when secondary dislocations are nucleated in the system at 8.6% strain. At this moment, a local rearrangement of the crystal lattice occurs, accompanied by a temporary rise in the dependence of vertical displacements on deformation.

The slip of GBs as whole dislocation walls is observed for systems with a Cu atom concentration in one column from 0 to 62.5% (see Table 1). In this case, there is an almost linear increase in the critical stress of plastic relaxation activation in the system (Figure 5e). In this case, a decrease in the flow stress occurs on the extended plateau, marked by two matching symbols “first minimum” and “second maximum”. The mechanism of plastic relaxation changes with a further increase in the Cu atom concentration in one column up to 75%.

### 3.2. Grain Rotation

**Figure 7:**
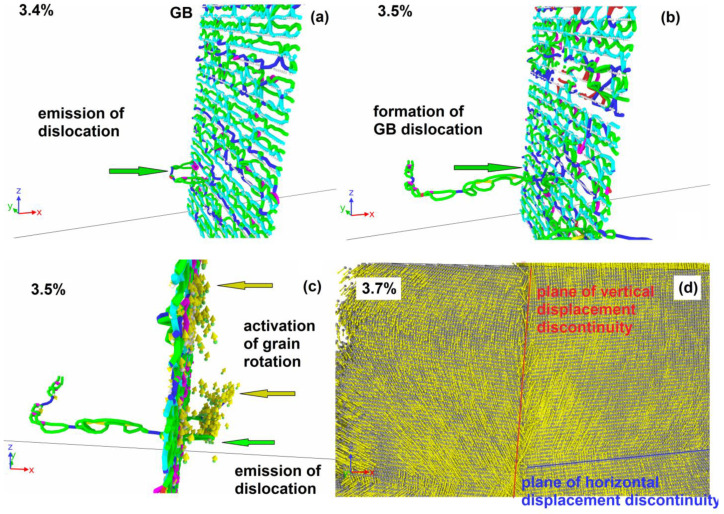
Deformation of system with 75% filling of one column by Cu atoms (3.85 nm^−2^). (**a**) Emission of dislocation semi-loop from GB into a grain. (**b**) Growth of dislocation semi-loop and formation of secondary dislocation loop at GB. (**c**) Emission of secondary dislocation loop into second grain and activation of grain rotation; vertically shifted atoms are shown with displacements. (**d**) Displacement vectors of atoms (yellow); in the left part of the panel, the displacements have a vertical component directed downwards, in the right part—upwards, the vertical displacements discontinuity is located between them (red line). In the right part of the system, traces of intragranular dislocation slip are visible, expressed as a discontinuity in horizontal atom displacements (blue line). Dislocation structure is determined by DXA: blue lines are perfect dislocations, green—Shockley partials, light blue—Frank partials, purple—stair rod segments.

In the 75% filing of one column system, plastic relaxation is activated when a dislocation is emitted from GB (Figure 7a). The dislocation segment is formed on the opposite of the place where several Cu atoms are missing in the column. The emitted dislocation consists of a different nature segment set and contains Shockley partials, perfect, and stair rod segments. In contrast to the case with a lower copper content, the separation of the entire dislocation line from the column does not occur, and after the removal of the moving segment deep into the grain, the GB dislocation is restored in the place from which the moving segment is emitted (Figure 7b). The dislocation forming the segment that closes the broken dislocation line in GB (Figure 7b) is a part of the loop (Figure 7c), the second half of which moves in the direction opposite to the segment (Figure 7a) emitted from the GB by the first. The dislocation emission from the GBs surface activates the grain rotation with sliding over GB surfaces. Figure 7d shows the field of vertical atom displacements on opposite sides of GBs, where a sharp discontinuity in the displacement direction is visible during the transition from one grain to another. Figure 7d shows the field of atom vertical displacements on opposite sides of GBs, where a sharp discontinuity in the displacement direction is visible with the transition from one grain to another. In this way, one can see a clear rotation of the grains in different directions. At the second GB, a similar process occurs. The slip traces of the emitted dislocation inside the grains are also visible in Figure 7d, which are expressed in the presence of a discontinuity in the horizontal displacement component. Further plastic deformation is accompanied by the development of both plasticity mechanisms.

The range of the grain rotation is observed for systems from the 75% filling of one column to 7.5% filling of three columns. Besides, the rotation of the entire system with the development of sliding only at one GB (see Table 1) is activated for the system 15% filling of three columns.

**Figure 8:**
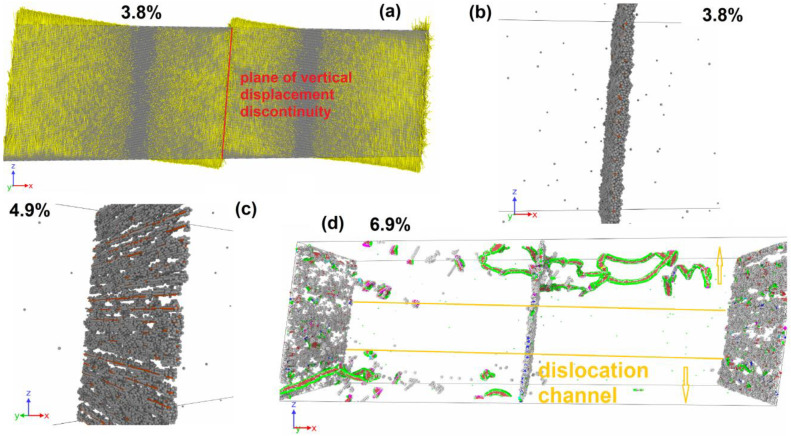
Deformation of system with 3.75% filling of three columns by Cu atoms (5.47 nm^−2^). (**a**) Displacement vectors of atoms (yellow); in each grain, the vertical displacements change sign; the vertical displacements discontinuity is located at both GBs (red line). Rotation center is visible in each grain. (**b**) Disordered layer near the GB along which the grains slide. Al atoms are grey dots, Cu—orange. Only atoms with central symmetrical parameter exceeding 5 are shown. (**c**) The same GB after rotation stopped. One can see the order of atoms on the GB (gaps appear in the defect atom distribution). (**d**) Formation of dislocation channel through both grains. Only atoms with central parameter exceeding 5 are shown. Grey dots are FCC-type atoms, red—HCP. Dislocation structure is determined by DXA: blue lines are perfect dislocations, green—Shockley partials, light blue—Frank partials, purple—stair rod segments.

The dependencies of stress, defect atom fraction, and vertical displacement on deformation are shown in Figure 5a,b,c for a system of 3.75% filling of three columns. The critical stress of plastic relaxation activation in this case reaches 2400 MPa at the strain of 3.8%. The stress–strain relationship shows a sharp drop in shear stresses after plastic relaxation activation (Figure 5a). Plastic relaxation in this case is activated due to the onset of grain rotation on both GBs. Figure 8a clearly shows sharp discontinuities in the vertical displacements of atoms located near GBs. In this case, there is no preliminary dislocation emission. With the beginning of grain boundary sliding, a layer of disordered atoms with a thickness of about 1.5 nm is formed, realizing the sliding of grains relative to each other (Figure 8b). The defect atom fraction (Figure 5b) and the vertical displacement (Figure 5c) increase sharply after the grains begin to rotate. The dependence of the defect atom fraction has a sharp peak, after which the defect atom number decreases. The maximum vertical displacement after a sharp increase reaches a near stationary level of 4 nm. Since the vertical displacement remains constant, the grain rotation stops, the layer of atoms on which the sliding occurs reduces its thickness as the atoms come to equilibrium positions, and the defect atom number decreases sharply (Figure 5b and Figure 8c). After the grain rotation stops, the stresses begin to increase again in the system. At the strain of 5.6%, dislocations are emitted from GBs and their number is not very large and does not provide an effective stress relaxation in the system. When the dislocation reaches the opposite GB, it is absorbed by the GB and then re-emitted into the neighboring grain. Thus, dislocation channels are formed in the grains, along which dislocation plasticity mainly occurs (Figure 8e). With the stresses increase in the system, starting from the strain of 6.8%, the grains rotate, and a small number of dislocations slip.

With the Cu concentration increases to 7.5 and 15% filling of three columns, there is a tendency for an earlier dislocation emission from GBs, immediately followed by rotation activation. Therefore, Table 1 lists two mechanisms for the plastic relaxation activation for these two systems. In Figure 5e, the indicated concentrations of Cu atoms correspond to area “2”, in which a smaller slope of the critical stress growth rate is observed in comparison with area “1”. Clear features are also visible in the dependencies of the first minimum and second maximum on the copper concentration.

### 3.3. Dislocation Emission 

**Figure 9:**
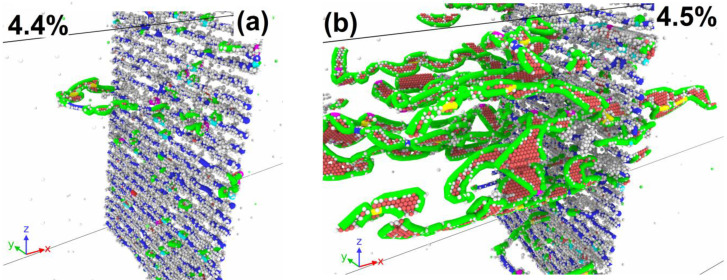
Deformation of system with 50% filling of three columns by Cu atoms (10.28 nm^−2^). (**a**) Emission of dislocation semi-loop from GB into a grain. Only atoms with central symmetrical parameter exceeding 5 are shown. (**b**) Intensive dislocation emission from GB. GB retains a structure with gaps between dislocations in the wall without the formation of a disordered layer. Only atoms with central symmetrical parameter exceeding 5 are shown. Grey dots are FCC-type atoms, red—HCP. Dislocation structure is determined by DXA: blue lines are perfect dislocations, green—Shockley partials, light blue—Frank partials, purple—stair rod segments.

At copper concentrations of 30–75% filling of three columns and 15–100% filling of five columns, plastic relaxation in the system is activated only due to the dislocation emission from GBs. Figure 9 shows two moments corresponding to the plastic relaxation onset and further development of plastic flow in the system with 50% filling of three columns. Initially, only GBs are the source of dislocations; however, with the development of deformation, the intragranular mechanisms of dislocation annihilation and multiplication are activated. The dislocation movement causes a vertical displacement in the system, but the rotation of the system’s parts does not develop.

In Figure 5d, these ranges of Cu content correspond to areas “3” and “5”. Critical stresses slightly increase; the first minimum shows a decline to almost zero in the area “3”. Critical stresses in area “5” are lower than the values achieved in areas “3” and “4” by 13% and change slightly with an increasing Cu content at GBs. 

### 3.4. Dislocation Emission and System Rotation 

In the Cu atom concentration range from 87.5% filling of three columns to 7.5% filling of five columns, plastic relaxation starts with the dislocation emission from GBs that leads to the rotation activation of the system as a whole with sliding at one of the GBs (Table 1).

**Figure 10:**
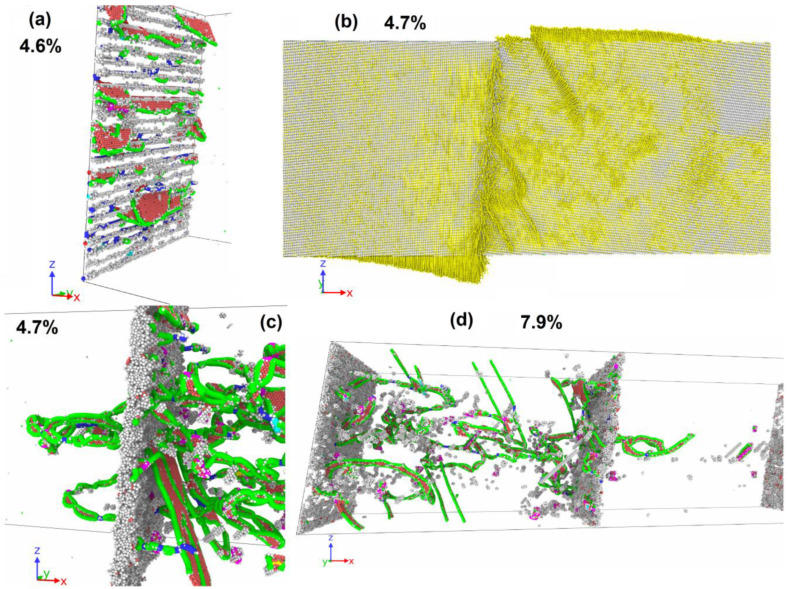
Deformation of system with 87.5% filling of three columns by Cu atoms (14.09 nm^−2^). (**a**) Emission of dislocations from GB into a grain. Only atoms with central symmetrical parameter exceeding 5 are shown. Grey dots are FCC-type atoms, red—HCP. (**b**) Displacement vectors of atoms (yellow); in the left part of the panel, the displacements have a vertical component directed downwards, in the right part, it is directed upwards, and the vertical displacements discontinuity is located between them. Traces of intragranular dislocation slip are visible, expressed as a discontinuity in both atom vertical and horizontal displacements. (**c**) Formation of a disordered layer at GB, which acts as a source of new dislocations. (**d**) Intragranular dislocation multiplication and emission from clusters of defect atoms in the left grain, emission of dislocations from GB into the right grain. Dislocation structure is determined by DXA: blue lines are perfect dislocations, green—Shockley partials, light blue—Frank partials, purple—stair rod segments.

Let us consider a system with 87.5% filling of three columns with Cu atoms as an example. Figure 10a shows the dislocation emission from GB at the strain of 4.5%. All emitted segments have the character of partial Shockley dislocations. The dislocation emission occurs only at one GB. After the emission of dislocations, the rotation of the system as a whole is activated with the development of sliding along one GB, which has acted as a source of dislocations. Figure 10b shows a GB with a clearly visible discontinuity in the vertical displacement of atoms on opposite sides of the GB. One can also see the traces of intragranular dislocation slip that activate the atom vertical displacement in the system. The dislocation emission from the GB leads to the formation disordered atoms layer near the GB, by which the sliding along the GB is realized (Figure 10c). The thickness of the formed disordered layer is about 1.5 nm. In Figure 5a, the activation of plastic flow leads to a sharp decrease in the stresses acting in the system. Note that this Cu atom concentration corresponds to the maximum critical stress achieved among all the cases considered. The defect atom number in the system increases sharply (Figure 5b) due to dislocation emission and the formation of a disordered atom layer near the sliding GB. After the initial vertical displacement increase (Figure 5c), the system rotation stops, and the atom vertical displacement remains close to constant. The atoms that formed the disordered layer, along which the sliding of the GB is realized, return in equilibrium positions that causes a decrease in the defect atom number (Figure 5b). In addition, the dislocation number in the system decreases, which makes a new stress increase possible after the strain of 5.5%. A small number of mobile dislocations exists in the system, until the strain reaches 7.8%; after that, the emission of new dislocations is activated from GBs, as well as from defects accumulated in the grains in the form of stair rod dislocations (Figure 10d).

The specified range of the Cu content corresponds to area “4” in Figure 5d. There, the maximum value of critical stresses is observed, after which the dependence of critical stresses on the Cu content has a drop and a following slight increase. In addition to the general mechanism of the onset of shear stress relaxation, the commonality of points can be seen in the dependences of the first minimum and second maximum on the copper concentration.

## 4. Discussion

According to our MD results, a tendency of Cu to segregate at GBs has been established: the Cu atom energy at GBs has been calculated to be equal to about −1 eV in comparison with the states inside the lattice remote from GB. This value is close in order of magnitude to the ab initio calculation results [17]. The decreased energy of Cu atoms at GBs facilitates their segregation at GBs as has been shown in the literature. All this makes relevant the investigation of the segregated Cu atom’s influence on the mechanical response of a material with GBs, which was elucidated in our MD study by the example of a symmetric tilt LAGB with the misorientation angle *θ* of about 12°, which is close to Σ99a <110>_Al_ GB type in the coincidence site lattice nomenclature [18]. In the experimental works, for instance, the results of torsion experiments [32] give an estimation of the Cu concentration in the range of 6–13 at.% in the GB region. In the present work, we considered the systems with the Cu content not exceeding the upper limit of this range and demonstrated that the upper limit of about 13 at.% corresponds to 5 completely filled columns of Cu atoms close to the center of a pentagonal GB void column; Cu atoms placed within these columns have the lowest energies.

Depending on the Cu content, the plastic relaxation mechanism changes from GB slip as the dislocation wall to the GB sliding and dislocation nucleation. At Cu contents at a GB below approximately 3.5 nm^−2^ (about 70% filling of one column), LAGBs slip (migrate) towards each other until annihilation. This scenario was previously investigated in detail in MD studies for pure metals [11,12,13]. In pure Al, the LAGB starts to slip at a certain level of shear stress of about 500 MPa, and this level is maintained during GB migration until annihilation. In the case of Cu-enriched GB, impurity atoms impede the GBs motion, and an additional peak of shear stress is formed before the activation of GB slip. The peak stress rises with the Cu content increase, and the LAGB separation from the Cu atoms is accompanied by stress relaxation down to about 500 MPa as in the pure aluminum case. An increase in the strength of NC aluminum with Cu segregation at the GB surface was recorded in many experimental works [27,28,29,30,31,32,33]. An increase in the GB strength was also observed in ab initio calculations for Cu segregation [17]. The ability of copper to prevent boundary slip, which is observed herein, can also explain the significant grain refinement leading to the formation of NC aluminum state, which was experimentally registered in [33].

The migration of LAGBs as dislocation walls leads to one of grain shrinkage and another grain growth, which is also the case even for the high-angle GBs [13] and for a complex grid of GBs with triple junctions [37]. Experimental evidence of the GB migration-induced growth and shrinkage of grains has been presented in the literature [38,39]. The LAGBs migration is terminated by their annihilation, which was also reported in previous MD simulations [11,12,13]. Annihilation takes place far from Cu atoms, but the annihilating GBs retain traces of detachment from Cu, and a more complex structure of immobile dislocation segments remains after the annihilation and influences on the following dislocation nucleation. Further deformation is elastic until dislocation nucleation from the annihilated GB remains. Even at this stage, the Cu atom’s presence in material decreases the nucleation threshold if compared with pure Al.

In pure metals, GB migration can be impeded by other GBs, triple junctions, or a high misorientation angle between grains [13]. In the considered case, Cu contents greater than about 3.5 nm^−2^ completely fix the GBs, and mechanisms of plastic relaxation different from migration are activated. The main mechanism is dislocations nucleation from the GB with segregated Cu atoms. In this case, the GB and additional defects related with Cu impurities decreases the threshold of dislocation nucleation in comparison with pure aluminum. The following dislocation plasticity can be supplemented by grain or system rotation. An alternative mechanism is the GB sliding activated in relatively narrow ranges of Cu content near the values of about 5 nm^−2^ and 15 nm^−2^. This mode of deformation produces a lower number of lattice defects in comparison with the dislocation plasticity and can be of interest for GB engineering. GB sliding leads to grain rotation, which is a well-known phenomenon in the literature [13,37], and can lead to GB elimination and grain coarsening/shrinkage through the reduction in the GB misorientation angle promoted by high temperatures or small grain sizes [40,41]. Our study shows that variation in the Cu content allows one to control the mechanisms and parameters of GB-related plastic relaxation, which can be a powerful tool for GB engineering.

## 5. Conclusions

1. Plastic relaxation mechanisms provided by a symmetric tilt LAGB with the misorientation angle *θ* of 12° and segregated Cu atoms with the maximal concentration of 13 at.% in the GB region are studied using MD simulations.
2. Cu segregation at GB most often leads to an increase in the critical resolved shear stress required to activate plastic relaxation that is consistent with experimental and computational works considering the effect of Cu segregation on macroscopic strength properties.
3. An increase in the Cu content at the GBs leads to a non-monotonic dependence of the critical resolved shear stress and change in plastic relaxation mechanisms.
4. Activation of GB sliding with grain rotation takes place in relatively narrow ranges of Cu content near the values of about 5 and 15 nm^−2^. This mode of deformation produces a lower number of lattice defects and can be of interest for GB engineering.
5. Complex interplay of plasticity mechanisms and non-monotonic variation in the flow stress with strain and the Cu content are the challenge for the theoretical description of this phenomenon.

## Figures and Tables

**Table 1:** Mechanisms of plastic relaxation and characteristic stresses for systems with different Cu content.

GB State	Copper Content (nm^−2^)	Mechanisms of Plasticity Activation	First Maximal Stress (MPa)	First Minimal Stress (MPa)	Second Maximal Stress (MPa)
Pure Al	0	GB slip	517	637	637
1 Cu column					
7.5	0.34	GB slip	763	603	603
15	0.81	GB slip	956	438	438
30	1.46	GB slip	1262	316	316
50	2.56	GB slip	1708	160	160
62.5	3.16	GB slip	2037	93	275
75	3.85	Dislocation emission from GBs + two grain rotation	2267	680	850
87.5	4.47	Two grain rotation	2294	689	1549
100	5.11	Two grain rotation	2320	672	1407
3 Cu columns					
3.75	5.47	Two grain rotation	2420	614	1435
7.5	5.87	Two grain rotation + dislocation emission from GBs	2560	489	1300
15	6.61	System rotation + dislocation emission from GBs	2725	300	1257
30	8.17	Dislocation emission from GBs	2879	220	736
50	10.28	Dislocation emission from GBs	2925	130	954
62.5	11.49	Dislocation emission from GBs	3000	104	678
75	12.8	Dislocation emission from GBs	3006	40	769
87.5	14.09	Dislocation emission from GBs + system rotation	3026	240	1065
95	14.82	Dislocation emission from GBs + system rotation	2982	252	910
100	15.32	Dislocation emission from GBs + system rotation	2614	221	1246
5 Cu columns					
1.88	15.51	Dislocation emission from GBs + system rotation	2516	252	1436
3.75	15.72	Dislocation emission from GBs + system rotation	2531	277	1094
7.5	16.08	Dislocation emission from GBs + system rotation	2640	209	1212
15	16.8	Dislocation emission from GBs	2720	427	869
50	20.48	Dislocation emission from GBs	2663	340	792
75	23.01	Dislocation emission from GBs	2707	269	618
100	25.53	Dislocation emission from GBs	2663	275	525

## Data Availability

Data available on request due to their large volume.
